# A Five-Year Study on Treatment Changes in Hypoglycemia-Associated Medications: Towards Personalized Diabetes Management

**DOI:** 10.3390/jpm16030150

**Published:** 2026-03-04

**Authors:** Amal Asiri, Indriastuti Cahyaningsih, Stijn de Vos, Jens H. J. Bos, Catharina C. M. Schuiling-Veninga, Eelko Hak, Sumaira Mubarik, Petra Denig, Katja Taxis

**Affiliations:** 1 Department of PharmacoTherapy, -Epidemiology, and -Economics, University of Groningen, 9713 AV Groningen, The Netherlands; i.cahyaningsih@rug.nl (I.C.); stijndev@gmail.com (S.d.V.); h.j.bos@rug.nl (J.H.J.B.); c.c.m.schuiling-veninga@rug.nl (C.C.M.S.-V.); e.hak@rug.nl (E.H.); s.mubarik@rug.nl (S.M.); k.taxis@rug.nl (K.T.); 2 Department of Pharmacy Practice, Faculty of Pharmacy, King Abdulaziz University, Jeddah 21589, Saudi Arabia; 3 Department of Pharmacist Profession Education, Faculty of Medicine and Health Sciences, Universitas Muhammadiyah Yogyakarta, Yogyakarta 55183, Indonesia; 4 Department of Clinical Pharmacy and Pharmacology, University of Groningen, University Medical Centre Groningen, P.O. Box 30.001, 9700 RB Groningen, The Netherlands; p.denig@umcg.nl

**Keywords:** hypoglycemia, treatment changes, glucose-lowering drugs, de-intensification, type 2 diabetes, primary care

## Abstract

**Background**: Understanding patient-specific patterns of medication intensification and de-intensification is essential for personalizing diabetes management and minimizing hypoglycemia risk in patients with type 2 diabetes. **Objectives**: To assess treatment changes in hypoglycemia-associated medications over five years and explore patient characteristics associated with these changes. **Methods**: We conducted a longitudinal cohort study using the IADB.nl database containing prescription data from Dutch community pharmacies. Individuals aged ≥35 years with at least two dispensations of glucose-lowering medications were identified. We estimated transition probabilities of changes in hypoglycemia-associated medications (sulfonylureas and/or insulin) using a Markov model for each year of follow-up. Associations with age, sex, and estimated hypoglycemia risk were explored with regression analysis. **Results**: Overall, 25,057 patients were included. Medication remained unchanged for the majority of the patients in the follow-up period. De-intensification increased from 4.7% (Year 1) to 6.5% (Year 5), while intensification decreased from 7.7% to 6.9% over the same period. Markov models showed that patients predominantly remained in a no change state over 5 years (transition probabilities: 0.92–0.94). High estimated hypoglycemia risk, age and being female were associated with intensification and/or de-intensification. **Conclusions**: While treatment regimens remained unchanged for most patients, de-intensification of hypoglycemia-associated medications increased modestly over five years. Factors like hypoglycemia risk, age and sex influenced changes. These findings support the need for personalized, risk-stratified approaches to diabetes medication management.

## 1. Introduction

Type 2 diabetes (T2D) is a progressive disease that requires periodic intensifications of treatment to maintain glycemic control and prevent microvascular and macrovascular complications [1]. However, intensive glucose-lowering therapies can increase the risk of hypoglycemia. This is particularly the case for patients on insulin or sulfonylureas, requiring to carefully balance the benefits and risks of the medication [2]. Strict glycemic control is generally not beneficial for patients with multiple comorbidities, older adults on polypharmacy, or those who are critically ill [3]. Therefore, de-intensification may be necessary, which involves reducing or stopping medication for patients at risk of hypoglycemia or when the benefits of intensive treatment no longer outweigh the risks [4]. Guidelines have been developed to assist healthcare providers in determining when de-intensification is appropriate, taking into account factors such as patient health status and the risk of adverse events [5]. Despite these guidelines, de-intensification occurs infrequently in clinical practice. De-intensification rates showed minimal variation across different hemoglobin A1c (HbA1c) levels [6]. Furthermore, barriers exist to deprescribing glucose-lowering treatments in older adults, and these need to be addressed to optimize care [7].

Assessing the risk of hypoglycemia is a crucial element in diabetes care, particularly in the primary care setting. A recently developed algorithm can be used to predict the hypoglycemia risk in T2D patients. The algorithm uses data from community pharmacies, including patient demographics and dispensing information. Primary care providers can use the risk score to identify patients who may benefit from treatment de-intensification [8,9]. A previous study applying this algorithm to a primary care dispensing database revealed that approximately 25% of patients with T2D may be at a high risk of hypoglycemia [10]. Among the patients with a high estimated risk, fewer than 9% received de-intensification of hypoglycemia-associated medications during a one-year follow-up period. Most high-risk patients experienced no changes in their medication regimens, highlighting a potential gap between guideline recommendations and clinical practice [5].

Personalized diabetes management requires moving beyond “one-size-fits-all” approaches to understanding how treatment patterns vary across patient subgroups. A wide variety of prescribing patterns with glucose-lowering medications have been found in drug utilization studies with longer periods of follow-up [11,12]. Moreover, patient characteristics, such as age and sex, can influence those prescribing patterns. For instance, studies have shown differences in treatment intensification timing, medication switching patterns, and adherence rates between male and female patients with T2D [13,14,15]. However, limited evidence exists on how treatment trajectories, particularly de-intensification patterns, vary over extended periods and how they are influenced by age, sex, and hypoglycemia risk. Understanding these patient-specific patterns can contribute towards developing tailored strategies that optimize glycemic control while minimizing hypoglycemia risk. Therefore, this study aimed to provide insights into individualized treatment modifications among T2D patients. The primary objective was to assess treatment changes in hypoglycemia-associated medications among patients over a five-year follow-up period. The secondary objective was to explore whether patient characteristics, such as hypoglycemia risk, age, and sex, are associated with these treatment modifications in a primary care setting.

## 2. Methods

### 2.1. Study Design and Setting

**Figure 1:**
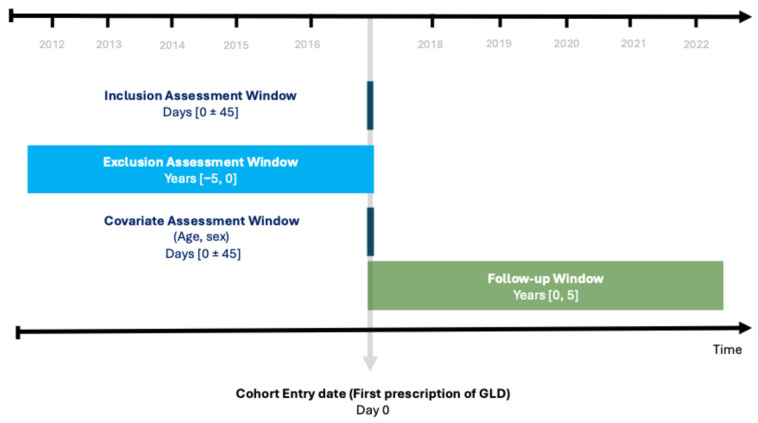
Study design diagram in calendar time. GLDs: glucose-lowering drugs.

A cohort of patients using medication indicated for T2D was followed for five years (2017–2022), as shown in Figure 1. For our analysis, we used the University of Groningen IADB.nl database, which is a comprehensive repository that collects and stores outpatient prescription information. This database aggregates prescription records from more than 125 community pharmacies operating across various regions of the Netherlands. This database, which has been used in many pharmaco-epidemiological studies, provides anonymized prescription records. This includes details, such as the Anatomical Therapeutic Chemical (ATC) codes of drugs, dates of dispensing, quantities, and daily dosages. The database does not contain information about medical diagnoses or medications administered during hospital stays. The study cohort is therefore based on dispensing information used as a proxy for the medical diagnosis. Studies using anonymized data in the Netherlands do not require ethical approval.

### 2.2. Study Population

The study cohort consisted of adults aged 35 years and older selected from the IADB.nl database. This is a database without diagnoses. We restricted to adults ≥35 years to lower misclassification of type 1 diabetes and gestational diabetes, as these conditions are more prevalent in younger adults and pregnancy, respectively. The index date was defined as the date of the first dispensing of any glucose-lowering medication (ATC code: A10) between 1 July 2016, and 30 June 2017. To restrict the cohort to prevalent users, we required ≥2 A10 dispensings within 365 days after the index date. The ≥2 dispensing requirement was applied to any glucose-lowering therapy and was not restricted to the same drug class. Patients receiving < 2 A10 dispensings in the first 365 days were excluded (spot users). Moreover, patients were required to have been registered in the IADB database for a minimum of 10 years, covering the five years prior to and five years following their index date (2011–2022). Patients were excluded if they had been prescribed insulin only (ATC code A10A) during the five-year period prior to their index date, as these individuals were likely to be diagnosed with type 1 diabetes. Additionally, data underwent screening to exclude any “dummy” patients included in the IADB.nl for testing purposes.

### 2.3. Outcome

The primary outcome measured was the change in hypoglycemia-associated medications over a five-year period from the index date. Glucose-lowering medications were divided into two categories based on the likelihood to induce severe hypoglycemia according to the Dutch guidelines for managing T2D [16]. The “hypoglycemia-associated” medications were sulfonylureas and insulins, which are known to carry a higher risk of severe hypoglycemic events than all other glucose-lowering drugs recommended in Dutch guidelines [16]. In this study, non-insulin glucose-lowering drugs include all glucose-lowering drugs except insulin (including sulfonylureas), whereas “other” glucose-lowering drugs exclude both sulfonylureas and insulin (Supplementary Table S1).

Treatment changes were categorized into three distinct groups.

A. No change: No modification of hypoglycemia-associated medication (sulfonylureas and/or insulin): (i) no modification of any glucose-lowering drug or (ii) only modifications of non-hypoglycemia-associated “other” medication.
B. Intensification: Addition of hypoglycemia-associated medication or switch to hypoglycemia-associated medication: (i) switch from non-insulin drugs (SU or other) to insulin, (ii) switch from other glucose-lowering drugs to sulfonylureas, or (iii) add sulfonylurea and/or insulin.
C. De-intensification: Discontinuation of or switch to medication not associated with hypoglycemia: (i) switch from insulin to non-insulin drugs (SU or other), (ii) switch from sulfonylureas to other glucose-lowering drugs, or (iii) discontinue sulfonylurea and/or insulin.

Operational definitions of glucose-lowering treatment changes are provided in Supplementary Table S1.

Treatment changes were evaluated over a five-year period using the anniversary method. To determine changes, we assessed prescribed medication each year starting with the index date. A ± 45 days eligibility window was applied at each assessment point to capture all dispensed medication in that period (index date and subsequent anniversary dates for Years 1–5). This specific window duration was selected based on prior sensitivity analyses that demonstrated its optimal performance in minimizing the misclassification of medication initiation and discontinuation, particularly for insulin therapy [10]. The anniversary method is particularly useful for tracking treatment changes in chronic conditions, such as T2D, as it provides a structured way to observe shifts in medication regimens and the introduction of new therapies over extended periods [17,18].

### 2.4. Statistical Analysis

The baseline demographic and clinical characteristics of the patients were summarized using descriptive statistics. Data were summarized as proportions for categorical variables, whereas continuous variables were presented as mean ± standard deviation (SD) for normal distributions and medians with interquartile ranges (IQRs) for skewed data.

We identified each patient’s status of medication changes for five consecutive annual intervals (index date to Year 1, Year 1 to Year 2, Year 2 to Year 3, Year 3 to Year 4, and Year 4 to Year 5). Changes were categorized as no change, intensification, or de-intensification, and we used descriptive statistics to summarize the frequency of each category at each interval. Moreover, we pooled all person-intervals across five years and identified specific subtypes within intensification and de-intensification categories based on operational definitions. Subtypes were ranked by frequency, and percentages were calculated within each category. To examine whether treatment change patterns varied by demographic characteristics, we calculated crude rates of no change, intensification, and de-intensification stratified by age groups (35–49, 50–64, 65–74, ≥75 years) and sex for each annual follow-up interval.

Next, we fitted a continuous-time multi-state Markov model with piecewise constant transition intensities over five years of follow-up. Markov states were defined annually as no change, intensification, and de-intensification. Clinically, a state should be interpreted as the net direction of regimen change during that year. Follow-up time was divided into consecutive one-year intervals using the anniversary method, with each patient’s medication state assessed at fixed annual time points following cohort entry. For each interval, the observed state was determined based on prescription changes occurring during that year. If multiple medication changes occurred within a single year, the state was defined as the final observed change within that interval. Intermediate changes within the year were not explicitly modeled. Transition probabilities were assumed to be constant within each one-year interval but allowed to vary across follow-up years (piecewise constant). Model parameters (transition intensities) were estimated by maximum likelihood. One-year transition probabilities for each interval t → t + 1 were then derived from the fitted model using the corresponding 1-year transition probability matrices. The Markov assumption, whereby transitions depend only on the current state and not on prior history, was considered reasonable given the annual aggregation of medication changes. Model fit was assessed by comparing observed and expected transition frequencies across follow-up years. Inclusion required continuous registration with complete dispensing capture over 5 years; therefore, registration gaps or incomplete patient-years were excluded by design.

Multinomial logistic regression was used to examine the association between the estimated hypoglycemia risk score, sex, and age at the index date and treatment modifications after five years from index date. The hypoglycemia risk score at the index date was calculated for each patient using the following variables: age, sex, total drug count at index date, count of glucose-lowering drugs (A10), sulfonylurea count (A10BB), insulin count (A10A), use of premixed insulin (A10AD), insulin count (A10A), use of antidepressants (N06A), and duration of insulin use (A10A) 5 years pre-index (Supplementary Table S2) [8]. The estimated risk score, ranging from 0 to 1, used a cutoff of 0.6 to identify patients at higher risk based on prior research [9]. Patients were then classified into two groups, low-risk (reference) and high-risk, according to their scores. Sex was treated as a categorical variable with being male as the reference group. Age was included in the model per steps of 10 years (based on age at the index date). Treatment outcomes were classified into no change (reference), intensification, or de-intensification of hypoglycemia-associated medications, by comparing the prescribing at the index date with prescribing at the end of Year 5. Because the hypoglycemia risk score incorporates age and sex, two multinomial logistic regression models were fitted to assess the impact of potential predictor overlap: Model 2 included hypoglycemia risk category only, and Model 1 additionally adjusted for age (per 10 years) and sex. The results were reported as odds ratios (ORs) with their corresponding 95% confidence intervals (95% CIs).

All statistical analyses were performed using R version 4.3.2 (R Foundation for Statistical Computing, Vienna, Austria) with msm and nnet packages. A two-sided *p* < 0.05 was considered statistically significant.

## 3. Results

### 3.1. Baseline Characteristics at the Index Date

**Figure 2:**
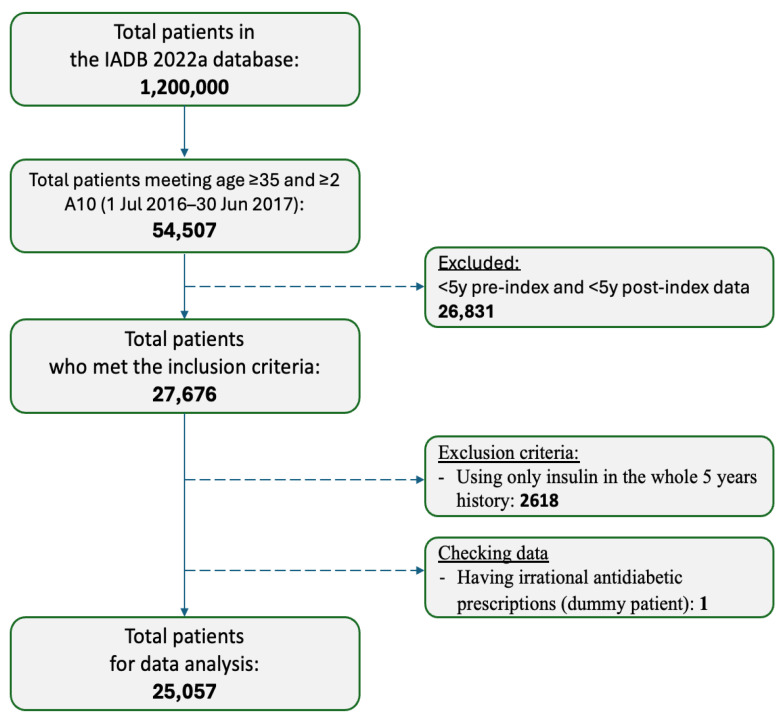
Flow chart of included patients.

A total of 25,057 T2D patients were included in this study (Figure 2). The mean age of the cohort was 66 years (SD: 11.11), with slightly more than half (51.8%) of the patients being male. Of the included patients, 25.9% had a high estimated hypoglycemia risk score. In total, 41.2% of patients were treated with metformin monotherapy, representing the most common non-hypoglycemia-associated regimen. In contrast, the most frequent hypoglycemia-associated regimen was a sulfonylurea in combination with non-insulin medication (26.4%), followed by insulin combined with other medication (15.5%) (Table 1).

### 3.2. Treatment Changes

Overall, most patients had no change in their hypoglycemia-associated medications, although this proportion declined slightly from 87.6% in the first year to 86.6% in the fifth year. The rate of intensification remained relatively stable (6.9–7.7%), while de-intensification increased from 4.7% to 6.5%. In the first year, 21,946 patients (87.6%) had no change, 1923 (7.7%) experienced intensification, and 1188 (4.7%) underwent de-intensification in hypoglycemia-associated medications. By the fifth year, the number of patients with de-intensification increased to 1618 (6.5%), whereas those with no change and intensification decreased to 21,710 (86.6%) and 1729 (6.9%), respectively (Table 2).

The most frequent intensification and de-intensification subtypes across all five follow-up years are presented in Table 3. Intensification was driven primarily by medication additions rather than switches. The most common pattern was the addition of SU to monotherapy (42.7%), followed by addition of insulin to combination therapy (22.6%). Addition of SU to combination therapy (10.6%) and addition of insulin to monotherapy (9.0%) were also notable contributors. De-intensification was dominated by medication discontinuations from combination therapy, particularly discontinuation of SU (49.2%) and insulin (30.8%). Monotherapy discontinuations were less frequent, accounting for 4.9% for SU and 3.3% for insulin (Table 3). Crude rates of treatment changes stratified by age groups and sex across five years were consistent with the overall pattern, with no change predominant across all years (Supplementary Table S3).

**Figure 3:**
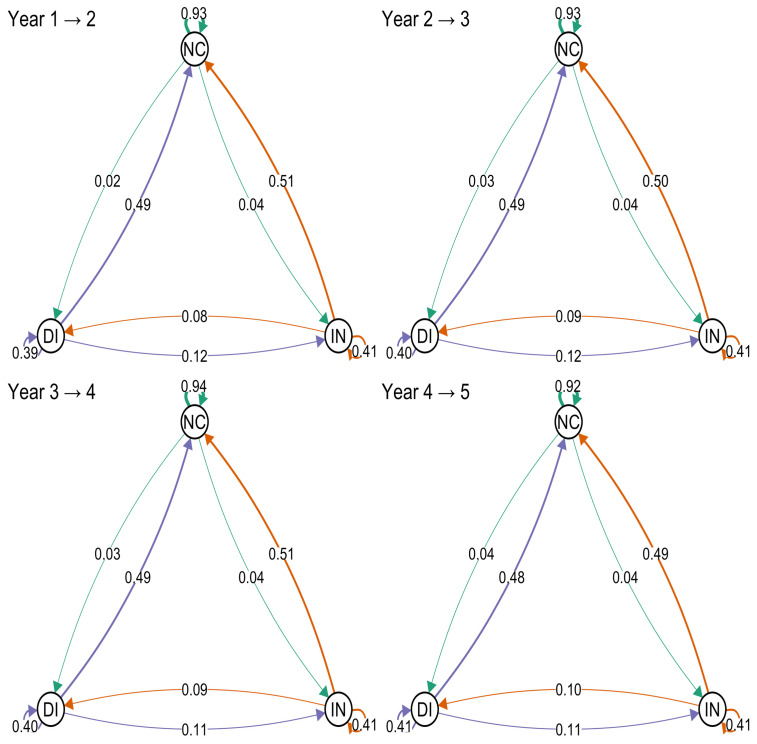
Network plots of 1-year transition probabilities among three treatment states across consecutive annual intervals (Years 1→2 through 4→5). Nodes represent no change (NC), intensification (IN), and de-intensification (DI). Directed edges are labeled with the estimated probability of transitioning from the origin state to the destination state; self-loops indicate remaining in the same state over the 1-year interval. Edge colors denote the origin state (green: NC; red: IN; blue: DI).

Annual 1-year transition probabilities between treatment change states were estimated from the fitted Markov model to summarize year-to-year patterns over follow-up (Figure 3). Across consecutive intervals, remaining in the no change state was most common (NC→NC: 0.92–0.94), whereas transitions from no change to intensification (NC→IN: 0.04–0.05) or de-intensification (NC→DI: 0.03–0.04) were infrequent. Among patients in intensification, persistence was moderate (IN→IN: ≈0.41), with most transitions returning to no change (IN→NC: 0.49–0.51) and fewer moving to de-intensification (IN→DI: 0.08–0.10). Similarly, persistence in de-intensification was ≈0.40–0.41 (DI→DI), with transitions most often returning to no change (DI→NC: 0.48–0.49) and less frequently moving to intensification (DI→IN: 0.11–0.12). For example, between Year 1 and Year 2, the probability of remaining in no change was 0.93, while probabilities of transitioning to intensification and de-intensification were 0.04 and 0.02, respectively. Corresponding 95% confidence intervals for all 1-year transition probabilities are provided in Supplementary Table S4.

After five years compared to the index state, medication of 16,765 (66.9%) patients was unchanged, 5314 (21.2%) intensified and 2978 (11.9%) de-intensified. An estimated high hypoglycemia risk score significantly increased the likelihood of de-intensification (OR 2.88, 95% CI 2.66–3.12) and markedly decreased the likelihood of intensification (OR 0.15, 95% CI 0.14–0.17). Per 10-year increase in age, the odds of intensification were lower (OR 0.80, 95% CI 0.78–0.82), whereas increase in age per 10 years was not significantly associated with de-intensification (OR 0.97, 95% CI 0.94–1.01). Additionally, being female was linked to increased odds of de-intensification (OR 1.12, 95% CI 1.03–1.21) but sex did not significantly influence intensification (OR 0.94, 95% CI 0.89–1.01). Associations for hypoglycemia risk category were similar in the risk-only model (Model 2) compared with the model additionally adjusted for age and sex (Model 1) (Table 4).

## 4. Discussion

### 4.1. Principal Findings

In this five-year longitudinal study of patients with T2D, we found that the majority had no changes in their hypoglycemia-associated medication regimens. There was a noticeable increase in de-intensification over time, rising from 4.7% in the first year to 6.5% by Year 5 whereas intensification remained relatively stable around 7%. The likelihoods of intensification and/or de-intensification appeared to be influenced by hypoglycemia risk score, age and female sex.

A relatively high proportion of patients experienced no change in hypoglycemia-associated medications in our cohort. This is in line with a large study conducted in the United States, which showed that between 2005 and 2014 the annual rates of intensification with sulfonylureas and with insulin were low (around 5.5 and 3.5 per 100 person-years, respectively) [19]. The overall proportion of 21.2% patients with an intensification after five years in our cohort seems also in line with the 25% of patients who progressed from metformin and/or sulfonylureas to insulin within 5 years after inclusion in a large trial [20]. Other studies have looked at shorter follow-up periods. A study in Spain showed that 6.3% of patients initially treated with metformin received an add-on with sulfonylureas and 6.6% with insulin within one year [21]. This apparent higher rate of treatment changes as compared to our findings may be explained by multiple factors, including variations in healthcare systems, local treatment guidelines and policies. Evidence from a range of European populations illustrated that the median time until a first treatment for T2D patients clearly varies across countries, with 37 months for the Netherlands as compared to 24 months for Spain, suggesting that regional prescribing practices and healthcare policies may indeed influence treatment stability [22].

The association of estimated hypoglycemia risk with more de-intensification after five years aligns with our prior findings, which showed a similar association after one-year follow-up [10]. These findings are also in line with other research, suggesting that patients with T2D who are at high risk of hypoglycemia are more prone to de-intensification of their treatment [23,24]. Furthermore, our study suggests that during follow-up, the chances of de-intensification slightly increase. However, the annual rate of de-intensification remains low, resulting in 11.9% of patients with de-intensified treatment observed after five years of follow-up. This finding is consistent with earlier research, which demonstrated that de-intensification of treatment among T2D patients is relatively uncommon [24,25,26]. Among older adults, somewhat higher de-intensification rates of glucose-lowering medication have been reported [4,27]. We did not find such an association in our study. However, we found a negative association of age (per 10-year increase) with treatment intensification which suggests that Dutch physicians may initiate fewer of those changes in older patients.

### 4.2. Strengths and Limitations

A strength is the analysis of long-term data from a large cohort of T2D patients treated in Northern Netherlands primary care practice. Furthermore, this study is unique in its extended follow-up period, which enabled us to evaluate the changes in treatments, particularly the de-intensification rates, in the T2D population. Another strength lies in our application of a pre-validated algorithm to evaluate hypoglycemia risk, specifically designed for T2D patients in primary care settings, which aligns with the context and population of our investigation. Finally, the use of a multi-state Markov model represents a unique approach to capturing treatment changes over time. This method provides insight into the patterns of treatment progression, allowing for varied transition probabilities across different time intervals.

This study has several limitations. First, our database lacks detailed clinical information, which introduces the possibility of misclassifying patients with type 1 diabetes as T2D. To minimize this misclassification, we excluded patients who had been dispensed insulin only before entering the cohort from the study. Phenotyping of patients was not possible, and therefore our cohort is heterogenous in this respect. More work is required to investigate the associations between particular phenotypes, medication use and hypoglycemia risk. This is particularly relevant for phenotype 5 which shows an increased risk to experience hypoglycemia with treatment [28]. Second, owing to difficulties in accurately capturing insulin dosing through pharmacy dispensing data, we were unable to include dose changes in our operational definitions. Medications were categorized based on their potential to cause hypoglycemia, as outlined in Dutch guidelines. Our definition of de-intensification is based on drug classes and may be clinically ambiguous. For example, a switch from insulin to a sulfonylurea is counted as de-intensification, even though sulfonylureas still carry a risk for hypoglycemia. Therefore, de-intensification should be interpreted as a move away from higher-risk therapy (especially insulin), not as an absence of hypoglycemia risk. Third, this study was an analysis of a pharmacy database based on dispensing data. Some data were not included, which could have provided relevant information such as blood glucose levels or HbA1c and comorbidities. These factors may explain why in some patients de-intensification or intensification was not conducted. Fourth, the use of a time window around the index and anniversary dates to identify dispensed medications may lead to potential misclassifications as medication may be de-intensified and subsequently intensified during these intervals. Our analysis thus captures only sustained changes in treatment. Additionally, the continuous-registration requirement of at least 5 years pre- and post-index excluded 26,831 (49.2%) patients. This may select a survivor cohort with more stable follow-up and dispensing capture, potentially limiting generalizability to patients with shorter observation times due to moving, changing pharmacies, or death. If the excluded patients differ systematically in morbidity or treatment dynamics, the estimated treatment change patterns may be biased. Finally, our cohort included both incident and prevalent users with varying durations of prior glucose-lowering therapy, and thus we did not account for how treatment history may influence transition probabilities. Future studies would employ a new-user design with an adequate wash-out period to evaluate its impact on regimen changes.

### 4.3. Implications for Practice and Research

This study contributes to our knowledge on treatment modifications in diabetes by identifying distinct age-, sex-, and risk-specific patterns in medication changes over time. Our findings demonstrate that treatment trajectories vary substantially across patient subgroups, with age and sex playing a role. These findings align with other evidence for moving beyond one-size-fits-all approaches to risk-stratified, individualized treatment strategies.

The need for tailored T2D treatment strategies is recommended to balance the risks and benefits of intensification and de-intensification while considering individual patient characteristics, phenotype, comorbidities, and frailty status [28,29,30,31,32]. This follow-up study enhances our knowledge of treatment change dynamics in T2D patients, particularly regarding de-intensification of hypoglycemia-associated medications. The de-intensification rate remained relatively low over the five-year follow-up period, suggesting that physicians may be cautious to de-intensify medications. Low de-intensification may also reflect practical barriers to deprescribing in primary care such as uncertainty about stopping medicines and fragmented care [33].

A Dutch intervention showed that community pharmacists performing tailored clinical medication reviews targeted at patients with a high risk of hypoglycemia can play an important role in deprescribing and medication management in people with T2D [9]. Patients were identified using a previously developed hypoglycemia risk algorithm based on routinely available dispensing data [8]. Further research on the benefits of integrating advanced risk prediction tools into clinical practices that support more proactive management of T2D is needed.

Longitudinal studies with long follow-up periods are important to provide deeper insights into the long-term effects of treatment changes and patient outcomes. Future research should utilize longitudinal modeling approaches to investigate long-term treatment patterns in diabetes management. Particularly, studies are required to evaluate personalized treatment strategies based on the risk of hypoglycemia, sex, age, and other patient-specific factors such as diabetes phenotype in various clinical settings. This method is particularly well suited to answer key questions, such as how treatment intensities evolve over time, which factors influence the timing and likelihood of transitioning between different treatment states, and how these transitions impact patient outcomes, such as the risk of hypoglycemia or the overall effectiveness of glucose control.

## 5. Conclusions

This five-year longitudinal study identified demographic and risk-specific patterns in hypoglycemia-associated medication changes among patients with type 2 diabetes. While treatment regimens remained unchanged for most patients, de-intensification increased modestly over time. Factors like hypoglycemia risk, age and female sex influenced changes. These findings support the need for personalized, risk-stratified approaches to diabetes medication management to balance glycemic control with hypoglycemia prevention across diverse patient populations.

## Figures and Tables

**Table 1:** Baseline characteristics of patients with T2D.

	Total	
	Number	%
Age (mean (SD)), years	66.19 (11.11)	
Sex		
Female	12,067	48.2
Male	12,990	51.8
Estimated hypoglycemia risk		
High risk	6492	25.9
Low risk	18,565	74.1
Therapy type for diabetes		
Monotherapy	11,932	47.6
Combination therapy	13,125	52.4
Treatment regimen types		
*Non-hypoglycemia-associated*		
Metformin monotherapy	10,315	41.2
Non-insulin, non-SU combination	437	1.7
DPP4 inhibitors monotherapy	47	0.2
GLP-1 analogs monotherapy	25	0.1
Alpha glucosidase inhibitors monotherapy	17	0.1
Thiazolidinediones monotherapy	10	0.0
SGLT2 inhibitors monotherapy	4	0.0
*Hypoglycemia-associated*		
SU + non-insulin combination	6623	26.4
Insulin + other combination	3886	15.5
Insulin + SU combination	1652	6.6
SU monotherapy	1075	4.3
Insulin combination	545	2.2
Insulin monotherapy	408	1.6
SU combination	13	0.1

Notes: T2D—Type 2 diabetes, SD—Standard deviation; SU—Sulfonylurea; DPP4—Dipeptidyl peptidase-4; GLP-1—Glucagon-like peptide 1; SGLT2—Sodium-glucose cotransporter-2.

**Table 2:** Annual transition matrices of hypoglycemia-associated medication changes (total number = 25,057).

	Begin State in Period	End State in Period		
Time Periods		No Change Number (%)	Intensification Number (%)	De-Intensification Number (%)
**Index to Year 1**	Subtotals in 1st period	21,946 (87.6%)	1923 (7.7%)	1188 (4.7%)
**Year 1 to Year 2**	No change	19,688	1480	778
	Intensification	1481	101	341
	De-intensification	833	324	31
	Subtotals in 2nd period	22,002 (87.8%)	1905 (7.6%)	1150 (4.6%)
**Year 2 to Year 3**	No change	19,860	1356	786
	Intensification	1433	88	384
	De-intensification	811	309	30
	Subtotals in 3rd period	22,104 (88.2%)	1753 (7.0%)	1200 (4.8%)
**Year 3 to Year 4**	No change	20,003	1247	854
	Intensification	1322	70	361
	De-intensification	860	310	30
	Subtotals in 4th period	22,185 (88.5%)	1627 (6.5%)	1245 (5.0%)
**Year 4 to Year 5**	No change	19,645	1345	1195
	Intensification	1191	64	372
	De-intensification	874	320	51
	Subtotals in 5th period	21,710 (86.6%)	1729 (6.9%)	1618 (6.5%)

**Table 3:** Most common subtypes of treatment change (intensification and de-intensification) across all years.

Category	Subtypes of Changes	Number	%
Intensification	Mono—Addition of SU	3818	42.7
	Comb—Addition of insulin	2022	22.6
	Comb—Addition of SU	951	10.6
	Mono—Addition of insulin	804	9.0
	Comb—Switching from non-SU to insulin	470	5.3
	Comb—Switching from SU to insulin	450	5.0
	Mono—Switching from non-SU to SU	252	2.8
	Comb—Switching from non-SU to SU	91	1.0
	Mono—Switching from non-SU to insulin	79	0.9
De-intensification	Comb—Discontinuation of SU	3148	49.2
	Comb—Discontinuation of insulin	1974	30.8
	Mono—Discontinuation of SU	311	4.9
	Comb—Switching from SU to non-SU	264	4.1
	Comb—Switching from insulin to non-SU	259	4.0
	Mono—Discontinuation of insulin	214	3.3
	Comb—Switching from insulin to SU	101	1.6
	Mono—Switching from SU to non-SU	95	1.5
	Mono—Switching from insulin to non-SU	22	0.3
	Mono—Switching from insulin to SU	13	0.2

Note: Mono = monotherapy at the start of the annual interval; Comb = combination therapy at the start of the annual interval. SU = sulfonylurea. Non-SU agents = non-insulin, non-sulfonylurea glucose-lowering drugs (metformin, DPP-4 inhibitors, SGLT2 inhibitors, GLP-1 receptor agonists, thiazolidinediones, α-glucosidase inhibitors).

**Table 4:** Patient characteristics influencing the treatment changes.

Factors		Odds Ratios (95%CI)	
		De-Intensification ǂ	Intensification ǂ
Model 1			
Hypoglycemia risk category	Low risk	Reference	Reference
	High risk	2.88 * (2.66–3.12)	0.15 * (0.14–0.17)
Sex	Male	Reference	Reference
	Female	1.12 * (1.03–1.21)	0.94 (0.89–1.01)
Age	Years §	0.97 (0.94–1.01)	0.80 * (0.78–0.82)
Model 2			
Hypoglycemia risk category	Low risk	Reference	Reference
	High risk	2.89 * (2.67–3.13)	0.16 * (0.14–0.18)

Note: ǂ Analyzed using multinomial logistic regression; no change as a reference outcome group. * Significant risk. § Odd ratios for age are per 10-year increase.

## Data Availability

The data that support the findings of this study are available from the IADB.nl prescription database (www.iadb.nl (accessed on 1 March 2026)). Access to the database is restricted and requires approval from the database administrators. The data are not publicly available due to privacy and data protection regulations.

## Supplementary Materials

The following supporting information can be downloaded at https://www.mdpi.com/article/10.3390/jpm16030150/s1, Table S1: Operational definitions of glucose-lowering treatment changes based on the risk of medication causing hypoglycemia; Table S2: The variables of the estimated hypoglycemia risk score; Table S3: Crude rate of treatment changes stratified by age and sex across 5 years; Table S4: One-year transition probabilities (95% CI) across follow-up.
