# Supplementary material for: A Five-Year Study on Treatment Changes in Hypoglycemia-Associated Medications: Towards Personalized Diabetes Management
Source: J Pers Med. 2026 Mar 4;16(3):150. doi: 10.3390/jpm16030150 (PMC13028196; doi:10.3390/jpm16030150)
Supplement: Supplementary file 1 [file jpm-16-00150-s001.zip › jpm-4069476-supplementary.pdf]

Supplementary Table S1. Operational definitions of glucose-lowering treatment changes based on the risk of medication causing hypoglycemia

Treatment changes	Monotherapy	Combination therapy
No Change	1. No modification of GLD.<br>- Insulin → insulin<br>- SU → SU<br>- Other GLD → other GLD<br>2. Addition/ discontinuation/ switch within other GLD only.	1. No modification of GLD(s).<br>- Insulin combination → insulin combination<br>- Insulin + SU → insulin + SU<br>- Insulin + other GLD → insulin + other GLD<br>- SU combination → SU combination<br>- Other GLD combination → other GLD combination<br>- SU + other GLD → SU + other GLD<br>2. Addition/ discontinuation/ switch within other GLD only.
Intensification	1. Switching non-insulin (SU or other GLD) to insulin.<br>2. Switching other GLD to SU.<br>3. Addition of SU or insulin.	1. Switching non-insulin (SU or other GLD) to insulin.<br>2. Switching other GLD to SU.<br>3. Addition of SU or insulin.
De-intensification	1. Switching insulin to non-insulin (SU or other GLD).<br>2. Switching SU to other GLD.<br>3. Discontinuation of SU or insulin.	1. Switching insulin to non-insulin (SU or other GLD).<br>2. Switching SU to other GLD.<br>3. Discontinuation of SU or insulin.

Notes

	Abbreviations	:	GLD, glucose-lowering drugs; SU, Sulfonylurea
1.	Index date	:	The date of the first prescription of GLD (ATC A10) between July 1, 2016, and June 30, 2017 with the time window (± 45 days from the index date).
	Follow-up period	:	The GLD (A10) prescribed within 5 years (2017-2022) after the index date, with the time window (± 45 days) around each anniversary date.
2.	Hypoglycemia-associated medication	:	Sulfonylureas, insulin (according to NHG guideline 2021).
3.	Other GLD	:	Metformin, Alpha glucosidase inhibitors, Thiazolidinediones, Dipeptidyl peptidase 4 (DPP-4) inhibitors, Glucagon-like peptide-1 (GLP-1) analogues, Sodium-glucose co-transporter 2 (SGLT2) inhibitors, other blood glucose-lowering drugs or fixed-dose combinations of any of these drugs, excl. insulins and sulfonylureas.
4.	Non-insulin GLD	:	All glucose-lowering drugs excluding insulin

Supplementary Table S2. The variables of the estimated hypoglycemia risk score

	High-risk		Low-risk	
	**n**	**%**	**n**	**%**
**Number of patients**	6,492		18,565	
**Risk score (median [IQR])**	0.84 (0.79-0.88)		0.39 (0.35-0.48)	
**Age (mean (SD)), years**ǂ	65.82 (11.13)		66.32 (11.11)	
**Sex**ǂ				
Female	3,219	49.6	8,848	47.7
Male	3,273	50.4	9,717	52.3
**Total drug count (median [IQR])** ǂ	9 [6-12]		6 [4-9]	
**Glucose-lowering drug count (median [IQR])**ǂ	3 [2-3]		1 [1-2]	
**Sulfonylurea count**ǂ				
0	4,838	74.5	10,858	58.5
1 or more	1,654	25.5	7,707	41.5
**Insulin count**ǂ				
0	2	0.0	18,564	100
1	3,632	55.9	1	0.0
2 or more	2,858	44.1	-	-
**Insulin duration (mean (SD)), years**ǂ	3.8 (1.4)		-	-
**Premixed insulin use**ǂ				
Yes	1,446	22.3	-	-
No	5,046	77.7	18,565	100
**Antidepressant use**ǂ				
Yes	968	14.9	2,282	12.3
No	5,524	85.1	16,283	87.7

Abbreviations: Sulfonylureas (SU)
Note: insulin use duration estimated over the five years prior to the index date.
All other variables estimated at the index date

Supplementary Table S3. Crude rate of treatment changes stratified by age and sex across 5 years

Year	Sex	Age group	No change n (%)	Intensification n (%)	De-intensification n (%)	Total
Year 1	Male	35–49	926 (83.9%)	114 (10.3%)	64 (5.8%)	1,104
		50–64	3,964 (85.7%)	431 (9.3%)	233 (5.0%)	4,628
		65–74	4,194 (88.6%)	338 (7.1%)	203 (4.3%)	4,735
		≥75	2,263 (89.7%)	139 (5.5%)	121 (4.8%)	2,523
	Female	35–49	818 (84.4%)	101 (10.4%)	50 (5.2%)	969
		50–64	3,096 (85.7%)	322 (8.9%)	195 (5.4%)	3,613
		65–74	3,614 (89.1%)	274 (6.8%)	170 (4.2%)	4,058
		≥75	3,071 (89.6%)	204 (6.0%)	152 (4.4%)	3,427
Year 2	Male	35–49	900 (81.5%)	133 (12.0%)	71 (6.4%)	1,104
		50–64	3,999 (86.4%)	387 (8.4%)	242 (5.2%)	4,628
		65–74	4,222 (89.2%)	325 (6.9%)	188 (4.0%)	4,735
		≥75	2,244 (88.9%)	184 (7.3%)	95 (3.8%)	2,523
	Female	35–49	795 (82.0%)	104 (10.7%)	70 (7.2%)	969
		50–64	3,108 (86.0%)	310 (8.6%)	195 (5.4%)	3,613
		65–74	3,628 (89.4%)	279 (6.9%)	151 (3.7%)	4,058
		≥75	3,106 (90.6%)	183 (5.3%)	138 (4.0%)	3,427
Year 3	Male	35–49	933 (84.5%)	119 (10.8%)	52 (4.7%)	1,104
		50–64	4,030 (87.1%)	377 (8.1%)	221 (4.8%)	4,628
		65–74	4,213 (89.0%)	318 (6.7%)	204 (4.3%)	4,735
		≥75	2,259 (89.5%)	146 (5.8%)	118 (4.7%)	2,523
	Female	35–49	810 (83.6%)	99 (10.2%)	60 (6.2%)	969
		50–64	3,145 (87.0%)	262 (7.3%)	206 (5.7%)	3,613
		65–74	3,622 (89.3%)	253 (6.2%)	183 (4.5%)	4,058
		≥75	3,092 (90.2%)	179 (5.2%)	156 (4.6%)	3,427
Year 4	Male	35–49	961 (87.0%)	86 (7.8%)	57 (5.2%)	1,104
		50–64	4,012 (86.7%)	388 (8.4%)	228 (4.9%)	4,628
		65–74	4,259 (89.9%)	274 (5.8%)	202 (4.3%)	4,735
		≥75	2,266 (89.8%)	135 (5.4%)	122 (4.8%)	2,523
	Female	35–49	821 (84.7%)	71 (7.3%)	77 (7.9%)	969
		50–64	3,138 (86.9%)	257 (7.1%)	218 (6.0%)	3,613
		65–74	3,630 (89.5%)	242 (6.0%)	186 (4.6%)	4,058
		≥75	3,098 (90.4%)	174 (5.1%)	155 (4.5%)	3,427
Year 5	Male	35–49	939 (85.1%)	95 (8.6%)	70 (6.3%)	1,104
		50–64	3,974 (85.9%)	354 (7.6%)	300 (6.5%)	4,628
		65–74	4,132 (87.3%)	299 (6.3%)	304 (6.4%)	4,735
		≥75	2,221 (88.0%)	149 (5.9%)	153 (6.1%)	2,523
	Female	35–49	806 (83.2%)	89 (9.2%)	74 (7.6%)	969
		50–64	3,083 (85.3%)	290 (8.0%)	240 (6.6%)	3,613
		65–74	3,561 (87.8%)	252 (6.2%)	245 (6.0%)	4,058
		≥75	2,994 (87.4%)	201 (5.9%)	232 (6.8%)	3,427

Supplementary Table S4. One-year transition probabilities (95% CI) across follow-up

Band	From	No changes	Intensification	De-intensification
Year 1→2	No changes	0.93 (0.93–0.93)	0.04 (0.04–0.05)	0.02 (0.02–0.03)
	Intensification	0.51 (0.49–0.53)	0.41 (0.40–0.43)	0.08 (0.07–0.08)
	De-intensification	0.49 (0.47–0.50)	0.12 (0.11–0.13)	0.39 (0.38–0.41)
Year 2→3	No changes	0.93 (0.93–0.94)	0.04 (0.04–0.04)	0.03 (0.02–0.03)
	Intensification	0.50 (0.49–0.52)	0.41 (0.39–0.43)	0.09 (0.08–0.09)
	De-intensification	0.49 (0.47–0.51)	0.12 (0.11–0.13)	0.40 (0.38–0.42)
Year 3→4	No changes	0.94 (0.93–0.94)	0.04 (0.04–0.04)	0.03 (0.03–0.03)
	Intensification	0.51 (0.49–0.52)	0.41 (0.39–0.42)	0.09 (0.08–0.10)
	De-intensification	0.49 (0.47–0.51)	0.11 (0.10–0.12)	0.40 (0.38–0.41)
Year 4→5	No changes	0.92 (0.92–0.93)	0.04 (0.04–0.04)	0.04 (0.03–0.04)
	Intensification	0.49 (0.48–0.51)	0.41 (0.39–0.42)	0.10 (0.09–0.11)
	De-intensification	0.48 (0.46–0.50)	0.11 (0.10–0.12)	0.41 (0.39–0.42)
